# Dacomitinib, a pan-inhibitor of ErbB receptors, suppresses growth and invasive capacity of chemoresistant ovarian carcinoma cells

**DOI:** 10.1038/s41598-017-04147-0

**Published:** 2017-06-23

**Authors:** Majid Momeny, Ghazaleh Zarrinrad, Farima Moghaddaskho, Arash Poursheikhani, Ghazaleh Sankanian, Azam Zaghal, Shahab Mirshahvaladi, Fatemeh Esmaeili, Haniyeh Eyvani, Farinaz Barghi, Zahra Sabourinejad, Zivar Alishahi, Hassan Yousefi, Reza Ghasemi, Leila Dardaei, Davood Bashash, Bahram Chahardouli, Ahmad R. Dehpour, Javad Tavakkoly-Bazzaz, Kamran Alimoghaddam, Ardeshir Ghavamzadeh, Seyed H. Ghaffari

**Affiliations:** 1 Haematology/Oncology and Stem Cell Transplantation Research Centre, Shariati Hospital, School of Medicine, Tehran University of Medical Sciences, Tehran, Iran; 2 Department of Medical Genetics, School of Medicine, Tehran University of Medical Sciences, Tehran, Iran; 3 Department of Molecular Systems Biology, Cell Science Research Centre, Royan Institute for Stem Cell Biology and Technology, ACECR, Tehran, Iran; 4 Section of Stem Cell Biology, Division of Oncology, Department of Medicine, Washington University in Saint Louis, Saint Louis, MO USA; 5 Massachusetts General Hospital Cancer Centre, Charlestown, MA USA; 6 Department of Haematology and Blood Banking, Faculty of Allied Medicine, Shahid Beheshti University of Medical Sciences, Tehran, Iran; 7 Experimental Medicine Research Centre, Tehran University of Medical Sciences, Tehran, Iran; 8 Department of Pharmacology, School of Medicine, Tehran University of Medical Sciences, Tehran, Iran

## Abstract

Epithelial ovarian cancer (EOC) is the most lethal gynaecological malignancy worldwide. Development of chemoresistance and peritoneal dissemination of EOC cells are the major reasons for low survival rate. Targeting signal transduction pathways which promote therapy resistance and metastatic dissemination is the key to successful treatment. Members of the ErbB family of receptors are over-expressed in EOC and play key roles in chemoresistance and invasiveness. Despite this, single-targeted ErbB inhibitors have demonstrated limited activity in chemoresistant EOC. In this report, we show that dacomitinib, a pan-ErbB receptor inhibitor, diminished growth, clonogenic potential, anoikis resistance and induced apoptotic cell death in therapy-resistant EOC cells. Dacominitib inhibited PLK1-FOXM1 signalling pathway and its down-stream targets Aurora kinase B and survivin. Moreover, dacomitinib attenuated migration and invasion of the EOC cells and reduced expression of epithelial-to-mesenchymal transition (EMT) markers *ZEB1*, *ZEB2* and *CDH2* (which encodes N-cadherin). Conversely, the anti-tumour activity of single-targeted ErbB agents including cetuximab (a ligand-blocking anti-EGFR mAb), transtuzumab (anti-HER2 mAb), H3.105.5 (anti-HER3 mAb) and erlotinib (EGFR small-molecule tyrosine kinase inhibitor) were marginal. Our results provide a rationale for further investigation on the therapeutic potential of dacomitinib in treatment of the chemoresistant EOC.

## Introduction

Epithelial ovarian cancer (EOC) is the fifth leading cause of cancer-related death among women worldwide and accounts for the highest mortality rate of all gynaecological malignancies. Each year, over 22000 women are diagnosed with EOC in the United States an estimated 14000 patients die from this disease^1^. Late-stage diagnosis, peritoneal metastasis and development of chemoresistance restrain improvements in overall survival rate. Despite debulking surgery and aggressive platinum/taxane-based chemotherapy regimens, the majority of patients relapse after achieving a complete clinical response^2, 3^. Inherent and acquired resistance to chemotherapeutics are responsible for treatment failure in EOC^4^. Patients with the recurrent disease are treated with gemcitabine and bevacizumab (anti-VEGFA mAb) but clinical trials report that the median overall survival is still dismal^5, 6^. Therefore, there is a pressing need to establish more effective therapies against chemoresistant EOC.

The ErbB or epidermal growth factor (EGF) family of receptor tyrosine kinases consists of four closely related members including EGFR, HER2, HER3 and HER4^7^. This family plays key roles in tumour growth, metastasis and therapy resistance through activation of down-stream pathways such as Ras/MAPK and PI3K/AKT^8, 9^. Evidence indicates that the ErbB family members are overexpressed in EOC which correlates with poor survival^10^. EGFR is overexpressed in 30–98% of EOC in all histologic subtypes^11, 12^. Enhanced expression of EGFR and its ligands correlate with advanced-stage disease, lack of therapeutic response and decreased recurrence-free survival^13–15^. *HER2* gene amplification and over-expression are found in different subtypes of EOC and associate with a higher recurrence frequency^16, 17^. Moreover, HER3 is up-regulated in EOC clinical samples which correlates with a worse prognosis^18, 19^.

The ErbB family is thought to drive malignant progression in EOC^20, 21^. EGFR and HER2 promote growth and chemoresistance^22, 23^. Moreover, HER3 and its ligand heregulin (HRG) play a central role in hematogenous dissemination of EOC cells to the omentum. HER3 is highly expressed in omental metastases in EOC patients and its knockdown impairs this organotropism *in vivo* ^24^. Altogether, these studies highlight the importance of the ErbB network in promoting growth, chemoresistance and metastatic progression in EOC and suggest that therapeutically disabling this family may hinder growth, survival and motility of EOC cells.

Despite the importance of EGFR in malignant progression in EOC, clinical trials with single-targeted agents such as cetuximab (anti-EGFR mAb), gefitinib and erlotinib (EGFR small molecule inhibitors) have demonstrated limited activity^25–27^. Trials of EGFR-directed therapies in combination with chemotherapeutics or other targeted therapies such as bevacizumab have also shown limited clinical benefit^28–30^. Furthermore, HER2-targeted therapies including trastuzumab and pertuzumab have proven to be of no benefit in EOC patients^31, 32^. Refinement of the ErbB-targeted therapeutics is therefore warranted to address resistance and maximize potential benefit in EOC patients.

Dacomitinib (Pfizer) is an irreversible second-generation pan-ErbB inhibitor^33^. A phase I study in patients with advanced solid tumours has demonstrated well-tolerated doses with significant antitumor activity^34^. Dacomitinib has shown promising clinical activity in advanced non-small cell lung cancer patients who progressed on platinum therapy and were previously treated with erlotinib^35, 36^. *In vitro* studies have reported significant anti-tumour activity of dacomitinib in gefitinib-resistant lung cancer as well as breast cancer cell lines which are resistant to trastuzumab and lapatinib (a dual HER2 and EGFR inhibitor)^37, 38^. In the present study, we examined the mechanistic activity of dacomitinib in chemoresistant EOC cells.

## Results

### Chemosensitivity of the EOC cell lines

The chemoresponsiveness of a panel of EOC cell lines to certain chemotherapeutics and targeted therapies were determined by MTT assay and are summarized in Table 1. These data show that OVCAR3, SKOV3 and A2780CP cells exhibit resistance to carboplatin, doxorubicin and cetuximab, as compared to A2780S and Caov4 cells (Table 1; Supplementary Fig. 1).

**Table 1:** Chemosensitivity of a panel of EOC cell lines to certain chemotherapeutics and targeted therapies.

Chemosensitivity (IC_50_)^a^								
Cell Lines	Cisplatin (μg/mL)	Carboplatin (μg/mL)	Paclitaxel (μg/mL)	Doxorubicin (ng/mL)	Vincristine (ng/mL)	Gemcitabine (ng/mL)	Erlotinib (μM)	Cetuximab (μg/mL)
OVCAR3	1.025	797.2	1.894	432.2	>1000	153.9	64.13	>100
SKOV3	5.799	71.32	5.355	696.1	>1000	24.14	113.6	>100
A2780CP	1.145	50.61	1.358	598.9	37.01	26.56	10.30	>100
A2780S	0.8634	4.594	0.2092	4.063	32.25	15.87	5.244	82.31
Caov4	0.3427	2.661	0.1155	5.102	3.430	4.560	2.635	43.89

Data represent the average of IC_50_ values and were collected from three independent experiments, each performed in triplicate. IC_50_ is the concentration of drug that caused a 50% reduction in proliferation compared to the vehicle-treated cells.

### Expression of the ErbB family in the EOC cells

**Figure 1:**
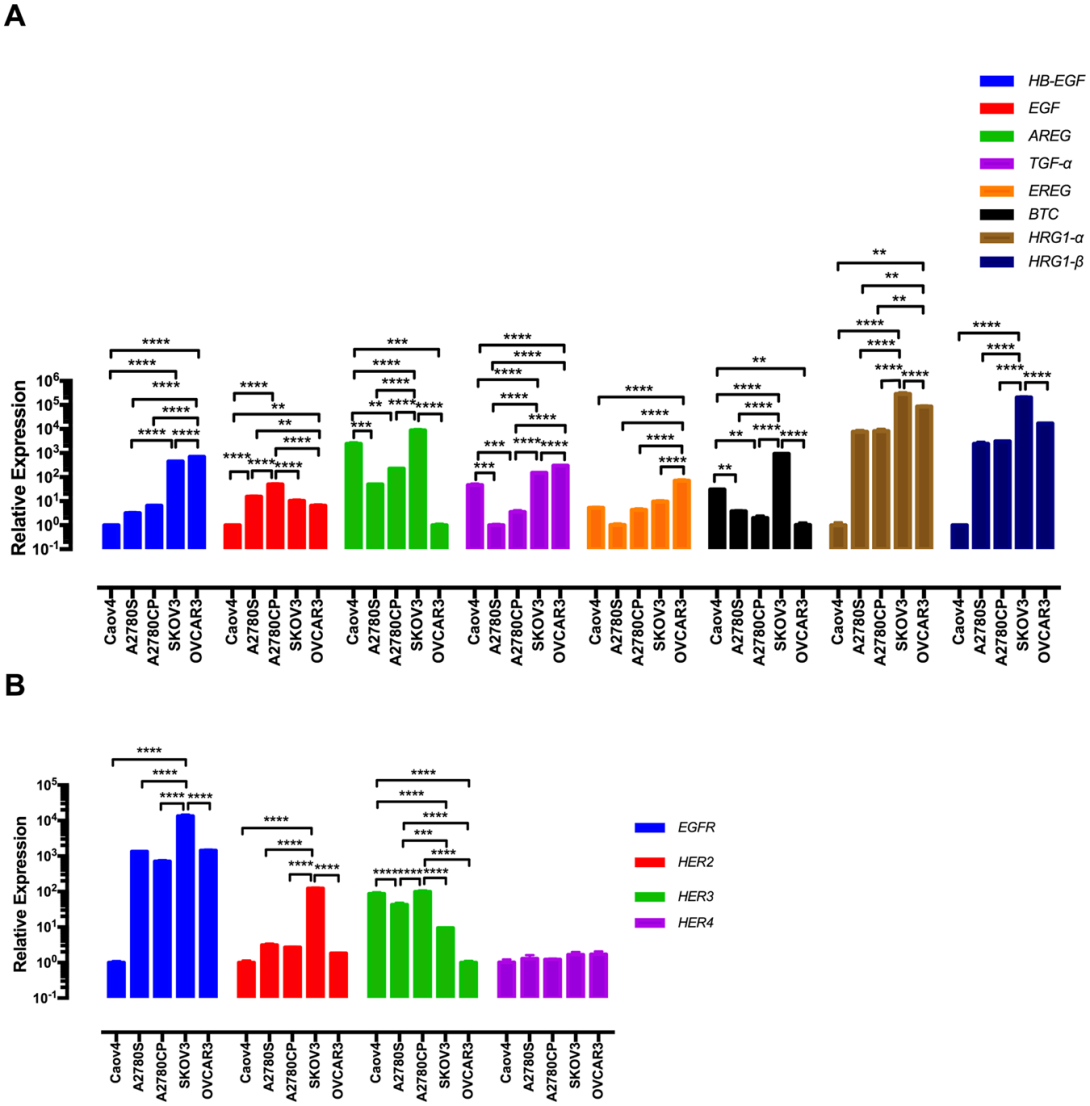
Expression of the ErbB family in the EOC cells. (**A**,**B**) The mRNA levels of the ErbB family in the EOC cell lines were determined by qRT-PCR. The data were evaluated in triplicate and collected from three independent experiments. Gene expression levels were normalised to *B2M* in each cell line. Data were analysed by one-way ANOVA followed by Tukey’s post hoc test and are shown as mean ± SD. Statistically significant values of *p < 0.05, **p < 0.01, ***p < 0.001 and ****p < 0.0001 were determined.

The expression of the ErbB family in chemoresistant versus sensitive EOC cells is not yet examined. To determine potential association between chemoresponsiveness and expression of the ErbB family, their relative expression was investigated by qRT-PCR. Using Tukey’s post hoc analysis, we found no difference in the expression of the ErbB family between the two groups of the EOC cells (Fig. 1A,B).

### The ErbB family contributes to cisplatin resistance

**Figure 2:**
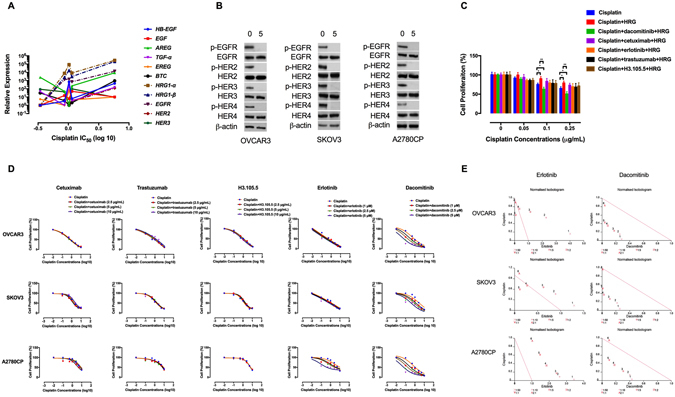
The ErbB family contributes to cisplatin resistance. (**A**) Correlation of expression of *HRG1*-*α*, *HRG1*-*β*, *EGFR* and *HER2* with resistance to cisplatin. EOC cell lines with higher expression of *HRG1*-*α*, *HRG1*-*β*, *EGFR* and *HER2* showed significantly higher cisplatin IC_50_ values. The correlation coefficient (r) between the expression of *EGFR* and *HER2* and cisplatin concentrations was 0.917 (*P* = 0.0281) and 0.890 (*P* = 0.0341), respectively. (**B**) Dacomitinib inhibits ErbB activation. The effect of dacomitinib (5 μM) on ErbB activation was determined by Western blot analysis. Protein lysates were subjected to Western blotting and probed with the indicated antibodies. β-actin was used as the loading control. The blots are representative of three independent experiments with similar results. (**C**) The effects of the ErbB inhibitors on HRGβ-1-induced proliferation in cisplatin-treated Caov4 cells were shown by MTT assay. The cells were pre-treated with the anti-ErbB agents for 4 h, followed by treatment with HRGβ-1 for 48 h. (**D**) The effects of the ErbB inhibitors-cisplatin therapy on cell proliferation were investigated by MTT assay after 48 h of treatment and the data are shown by IC_50_ shift analysis. The concentrations of cisplatin were 0.1, 0.5, 1, 2.5, 5 and 10 μg/mL. (**E**) Normalised isobolograms of combination of erlotinib (5 μM) and dacomitinib (5 μM) with cisplatin. The data were analysed using the CalcuSyn software. The connecting line represents additivity. Data points located below the line indicate a synergistic drug-drug interaction and data points above the line indicate an antagonistic interaction. The numbers under the isobolograms indicate the concentrations of the drugs in combination. Data shown represent the mean ± SD from three independent experiments, each performed in triplicate. Statistically significant values of *p < 0.05 and **p < 0.01 were determined compared with the control.

In an attempt to examine possible correlation between the mRNA levels of the ErbB family and chemoresponsiveness, we found that higher expression of *HRG1*-*α* and *HRG1*-*β* are significantly associated with resistance to cisplatin by Pearson’s correlation (Fig. 2A). The correlation coefficient (r) between the expression of *HRG1*-*α* and *HRG1*-*β* and cisplatin IC_50_ values is 0.9058 (*P* = 0.034) and 0.8997 (*P* = 0.037), respectively. In addition, our data demonstrated positive correlation between cisplatin resistance and higher expression of *EGFR* and *HER2* (Fig. 2A). We found no significant association between the ErbB family expression and resistance to carboplatin, paclitaxel, doxorubicin, gemcitabine and erlotinib (Supplementary Fig. 2).

Expression of both HRG1-α and HRG1-β has been observed in 30–83% of EOC cell lines and tumour samples^39^. After binding to HRG, HER3 heterodimerises with the other ErbB receptors, which activates down-stream pro-survival pathways^40^. HRG1-β binds to HER3 with more affinity and induces greater activation of the ErbB receptors than HRG1-α^41, 42^. We therefore explored the effects of exogenous HRGβ-1 on proliferative response of the chemosensitive Caov4 cells to cisplatin. The resulting data show that pre-treatment with HRGβ-1 (10 ng/mL) decreased cisplatin-induced cytotoxicity, a process abrogated when the cells were pre-treated with dacomitinib but not single-targeted ErbB inhibitors including cetuximab, erlotinib, trastuzumab and H3.105.5 (a ligand-blocking anti-HER3 antibody) (Fig. 2C).

We next compared the effects of the ErbB inhibitors on potentiation of the anti-tumour effects of cisplatin in the chemoresistant EOC cells. Combination of dacomitinib with cisplatin had a synergistic effect on growth inhibition. In comparison, erlotinib-cisplatin therapy was antagonistic (Fig. 2D,E; Supplementary Tables 1, 2). Altogether, these findings suggest that the ErbB family might contribute to cisplatin resistance and a pan-ErbB inhibition strategy is required to augment cisplatin efficacy in the chemoresistant EOC cells.

### Dacomitinib inhibits cell viability and induces apoptosis

**Figure 3:**
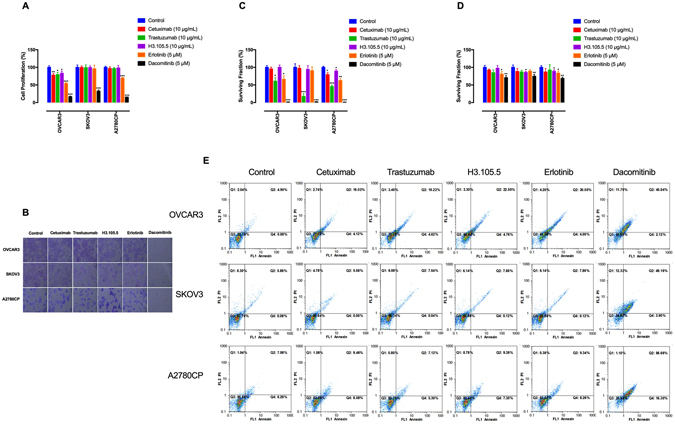
Dacomitinib inhibits cell growth and induces apoptosis. (**A**) The effects of the ErbB inhibitors on cell proliferation were estimated by MTT assay after 48 h of treatment. (**B**) The effects of the anti-ErbB agents on cell viability were demonstrated by crystal violet staining. The cells were treated with the ErbB inhibitors for 48 h, then stained with crystal violet and imaged by an inverted microscope (images acquired at 10x magnification). (**C**) Clonogenic assay was conducted to evaluate the effects of the ErbB inhibitors on clonal proliferation. (**D**) Anoikis resistance assay was performed with cell culture on poly-HEMA–coated culture dishes for 48 h and the proportion of viable cells was measured by MTT assay. (**E**) The effects of the anti-ErbB agents on induction of apoptosis were determined by annexin V staining. Annexin V-positive cells are considered early apoptotic, whereas PI uptake indicates necrosis. Cells positive for both stains are considered late apoptotic. The graphs are representative of three independent experiments with similar outcomes. Data shown represent the mean ± SD from three independent experiments, each performed in triplicate. Statistically significant values of *p < 0.05, **p < 0.01, and ***p < 0.001 were determined compared with the control.

MTT assay was performed to estimate anti-proliferative effects of the anti-ErbB agents on the chemoresistant EOC cells. Treatment with dacomitinib inhibited cell growth (Fig. 3A,B). Clonogenic capacity represents the renewal potential and a long-term response of cells after treatment. The results of a colony formation assay demonstrate that both trastuzumab and dacomitinib reduced clonogenic survival (Fig. 3C). In immortalized cells, detachment from the extracellular matrix induces anoikis, a special type of apoptosis^43^. Acquisition of resistance to anoikis is a prerequisite for EOC cells to survive in ascites before forming metastatic foci^44^. Our data show that dacomitinib diminished anoikis resistance (Fig. 3D). Moreover, dacomitinib but not the single-targeted agents induced apoptotic cell death, as demonstrated by Annexin V staining (Fig. 3E). These data suggest that dacomitinib had stronger anti-proliferative efficacy compared to the single-targeted ErbB inhibitors (Fig. 3A–E).

### Dacomitinib inhibits PLK1-FOXM1 signalling

Polo-like kinase 1 (PLK1) is a serine/threonine protein kinase which plays a central role in mitotic progression and its elevated expression in EOC correlates with histological grade^45^. PLK1 induces forkhead box protein M1 (FOXM1), a member of FOX family of transcription factors that regulates expression of a wide range of genes such as *PLK1*, survivin (encoded by *BIRC5*), cyclin B1 (encoded by *CCNB1*) and Aurora kinase B (encoded by *AURKB*)^46, 47^. The FOXM1-target genes participate in different cellular functions including cell growth, metastatic dissemination and therapy resistance^48, 49^.

**Figure 4:**
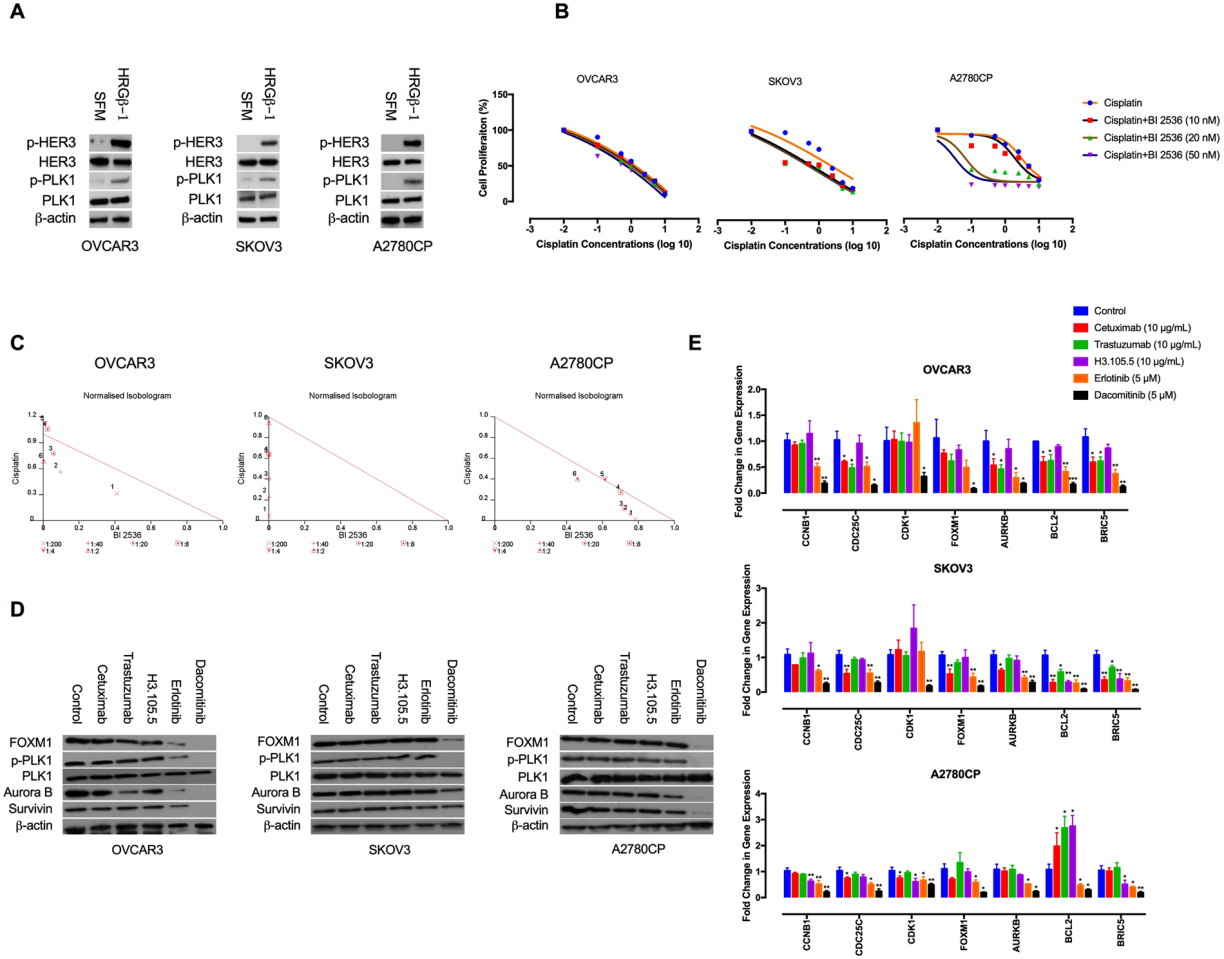
Dacomitinib inhibits PLK1-FOXM1 pathway. (**A**) HRG/HER3 loop activates PLK1. The effects of HRGβ-1 on PLK1 activation was determined by Western blot analysis. Protein lysates from serum-starved and HRGβ-1-treated cells were subjected to Western blotting and probed with the indicated antibodies. The blots are representative of three independent experiments with similar outcomes. (**B**) PLK1 blockade increases cisplatin sensitivity. The effects of BI 2536-cisplatin therapy on cell proliferation were investigated by MTT assay and shown by IC_50_ shift analysis. The cultures were treated with BI 2536 (20 nM) and cisplatin (0.1, 0.5, 1, 2.5, 5 and 10 μg/mL) for 48 h. (**C**) Normalised isobolograms of combination of BI 2536 and cisplatin. (**D**) The effects of cetuximab (10 μg/mL), trastuzumab (10 μg/mL), H3.105.5 (10 μg/mL), erlotinib (5 μM) and dacomitinib (5 μM) on PLK1-FOXM1 pathway and its down-stream targets were determined by Western blot analysis. The blots are representative of three independent experiments with similar outcomes. (**E**) The effects of the anti-ErbB agents on the expression of PLK1-FOXM1 targets genes were determined by qRT-PCR analysis. Data shown represent the mean ± SD from three independent experiments, each performed in triplicate. Statistically significant values of *p < 0.05, **p < 0.01, and ***p < 0.001 were determined compared with the control. SFM, serum-free media; CCNB1, cyclin B1; CDC25C, cell division cycle 25C; CDK1, cyclin-dependent kinase 1; FOXM1, Forkhead box M1; AURKB, Aurora kinase B; BIRC5, survivin.

PLK1 has been shown to mediate resistance to chemotherapeutics including cisplatin^50^. To determine if the ErbB family activates PLK1 in the EOC cells, the cells were serum-starved for 24 h and then treated with HRGβ-1 (10 ng/mL) for 30 min. Immunoblotting analysis showed that HRGβ-1 stimulation resulted in activation of PLK1 (Fig. 4A). This is in consistency with previous reports that the ErbB receptors activate the PLK1-FOXM1 axis^51, 52^. We next sought if dacomitinib-induced sensitisation to cisplatin is through inhibition of PLK1 pathway. To achieve this, the cells were treated with cisplatin in combination with BI 2536, a highly selective PLK1 inhibitor. Our findings demonstrate that BI 2536 had synergistic activity with cisplatin on inhibition of cell growth (Fig. 4B,C; Table 2), suggesting that PLK1 blockade enhances sensitivity to cisplatin.

**Table 2:** Combination index (CI) and dose reduction index (DRI) of BI 2536 and cisplatin combination in OVCAR3, SKOV3 and A2780CP cells.

Concentrations		fa	CI	DRI	
BI 2536 (nM)	Cisplatin (μg/mL)			BI 2536	Cisplatin
**OVCAR3**					
20	0.1	0.23	0.724	2.442	3.181
20	0.5	0.42	0.658	10.405	1.780
20	1	0.51	0.832	17.559	1.290
20	2.5	0.64	1.083	41.129	0.944
20	5	0.75	1.136	100.142	0.888
20	10	0.90	0.693	529.397	1.448
**SKOV3**					
20	0.1	0.45	0.049	424.093	21.290
20	0.5	0.46	0.229	624.218	4.393
20	1	0.49	0.402	2972.447	2.491
20	2.5	0.62	0.640	7.8e + 005	1.562
20	5	0.79	0.627	5.39e + 009	1.594
20	10	0.84	0.944	1.83e + 011	1.060
**A2780CP**					
20	0.1	0.55	0.797	1.278	70.538
20	0.5	0.57	0.818	1.327	15.428
20	1	0.59	0.836	1.389	8.592
20	2.5	0.6	0.974	1.426	3.661
20	5	0.66	1.012	1.629	2.511
20	10	0.76	0.863	2.172	2.483

DRI represents the order of magnitude of dose reduction that is allowed in combination for a given degree of effect as compared with the dose of each drug alone. “fa” denotes fraction affected.

We therefore evaluated the effects of dacomitinib on PLK1-FOXM1 signalling. Our data show that dacomitinib, but not the single-targeted agents, inhibited p-PLK1 (Fig. 4D). Furthermore, dacomitinib reduced the mRNA and protein levels of FOXM1, survivin and Aurora kinase B. Conversely, the inhibitory effects of the single-targeted agents were marginal (Fig. 4D,E). Altogether, these data suggest that PLK1 blockade is a mechanism for dacomitinib-induced sensitisation to cisplatin and that a comprehensive ErbB inhibition strategy is required for blockade of PLK1-FOXM1 pathway and its down-stream targets.

### Dacomitinib reduces migration and invasion

**Figure 5:**
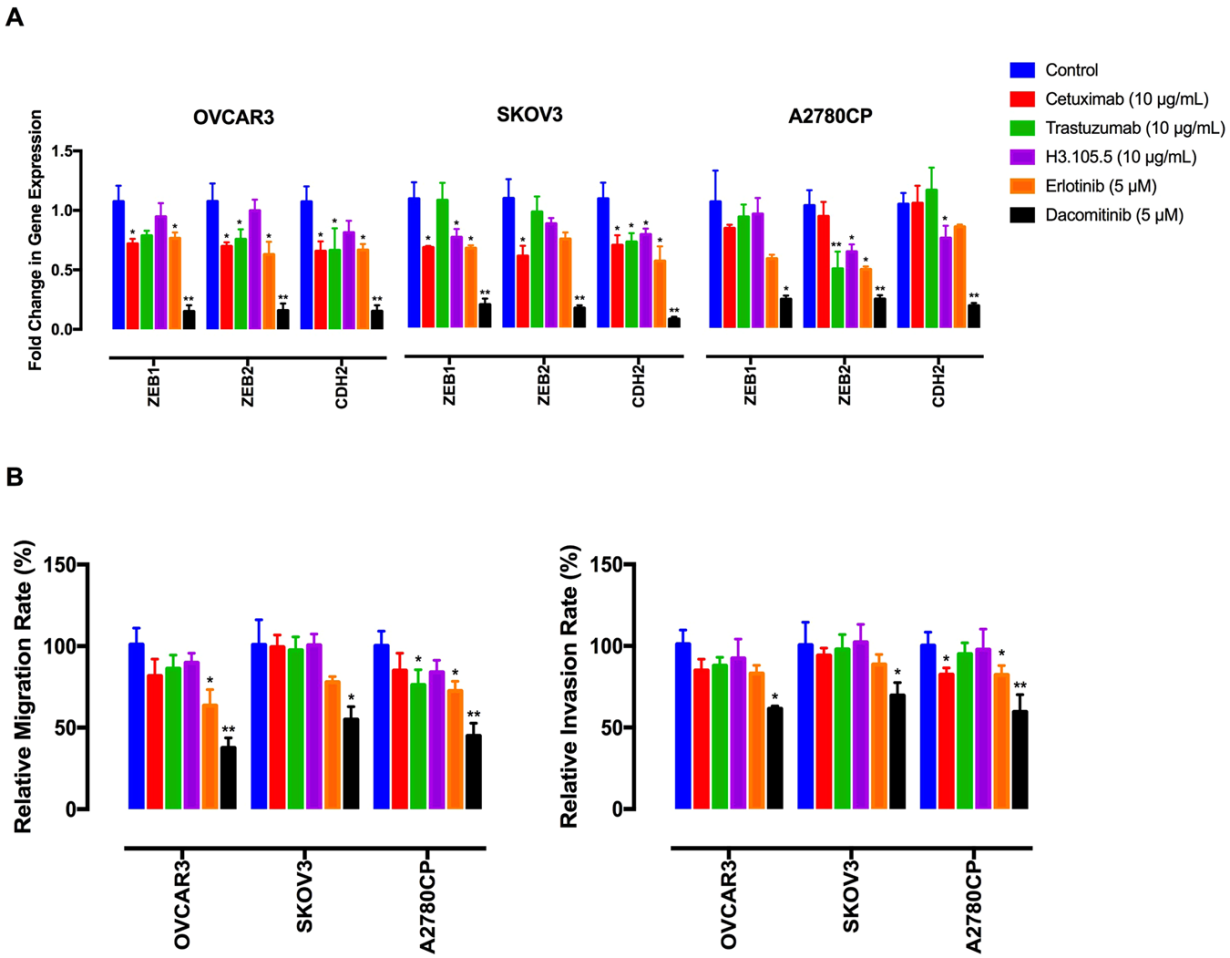
Dacomitinib inhibits migration and invasion. (**A**) The effects of the anti-ErbB agents on expression of EMT markers were determined by qRT-PCR analysis. (**B**) The effects of the ErbB inhibitors on cell migration and invasion. The cells were placed into 8-μm porous culture inserts, treated with the drugs and allowed to migrate for 48 h. The migrated cells on the lower surface of the inserts were quantified by crystal violet staining. For invasion assay, the cells were placed into matrigel-coated inserts and allowed to invade through the matrigel layer for 48 h. Data shown represent the mean ± SD from three independent experiments, each performed in triplicate. Statistically significant values of *p < 0.05 and **p < 0.01 were determined compared with the control.

Ovarian cancer metastasis includes tumour cells detachment from the primary site followed by their spread to the peritoneum and omentum^53^. The degree of peritoneal dissemination associates with poor prognosis^54^. Detachment of EOC cells from the primary site and their local invasion is driven by an epithelial-to-mesenchymal transition (EMT)^55^. EMT is triggered by down-regulation of cell adhesion molecules by transcriptional repressors ZEB1, ZEB2 and Snail^56^. It is thought that EMT contributes to loosening of intercellular adhesions and shedding of EOC cells into ascites^57^. We next determined the effects of dacomitinib on expression of the EMT markers *ZEB1*, *ZEB2* and *CDH2* (which encodes N-cadherin). The resulting data indicate that dacomitinib had stronger inhibitory effects on the expression of the EMT markers, as compared to the single-targeted agents (Fig. 5A). Moreover, these data show that dacomitinib hindered migration and invasion of the EOC cells through matrigel (Fig. 5B).

## Discussion

There is evidence that the ErbB signalling network contributes to chemoresistance in EOC. EGFR drives resistance to cisplatin through induction of the anti-apoptotic protein Bcl-2^58^. HER2 promotes resistance to taxane chemotherapies and its depletion enhances chemosensitivity^59^. Moreover, activation of HER3 has been demonstrated to drive chemoresistance in EOC cells via activation of AKT pathway^60^. These findings suggest that the ErbB family is a potential therapeutic target in the chemoresistant EOC and its blockade might inhibit tumour growth and induce chemosensitisation^61^.

During ovarian carcinoma metastasis, epithelial cells lose E-cadherin-mediated cell-cell interactions, up-regulate N-cadherin and undergo EMT^62^. Evidence indicates that EMT correlates with a poor prognosis in EOC^63, 64^. Moreover, EMT is thought to drive a chemoresistant behaviour^65, 66^. Induction of EMT promotes peritoneal dissemination and reversing the EMTed phenotype is believed to be a novel strategy to hamper intraperitoneal metastasis^67^. Targeting signalling pathways contributing to EMT is a potential therapeutic approach in order to hinder invasiveness of EOC cells^68^. Both EGFR and HER2 downregulate E-cadherin expression, induce an EMTed phenotype and increase motility of EOC cells^69, 70^. The results of the present study suggest that blocking the ErbB receptors by dacomitinib is an effective strategy in order to reduce the expression of the EMT markers and hamper invasive capability of the chemoresistant EOC cells.

Single-targeted ErbB agents have shown minimum response in chemoresistant ECO patients^26, 31, 71^. Compensatory activation of the other ErbB receptors sustains the activation of common downstream pro-survival pathways^72^. Targeting all the ErbB receptors is therefore a more effective treatment strategy, especially when resistance to a single-targeted ErbB agent has occurred^73^. For instance, breast cancer patients who experienced tumour progression after treatment with trastuzumab have demonstrated response to the dual EGFR and HER2 inhibitor lapatinib^74^. Furthermore, cetuximab-resistant colorectal and head and neck squamous cell carcinoma cells are sensitive to pan-ErbB inhibitors^75–77^. In consistency, our data show that the inhibitory effects of the single-targeted ErbB inhibitors on viability and invasiveness of the chemoresistant EOC cells were marginal. Conversely, dacomitinib exerted pronounced anti-tumour activity, suggesting that it may have potential for treatment of the EOC patients who ultimately have developed resistance after initial response.

**Figure 6:**
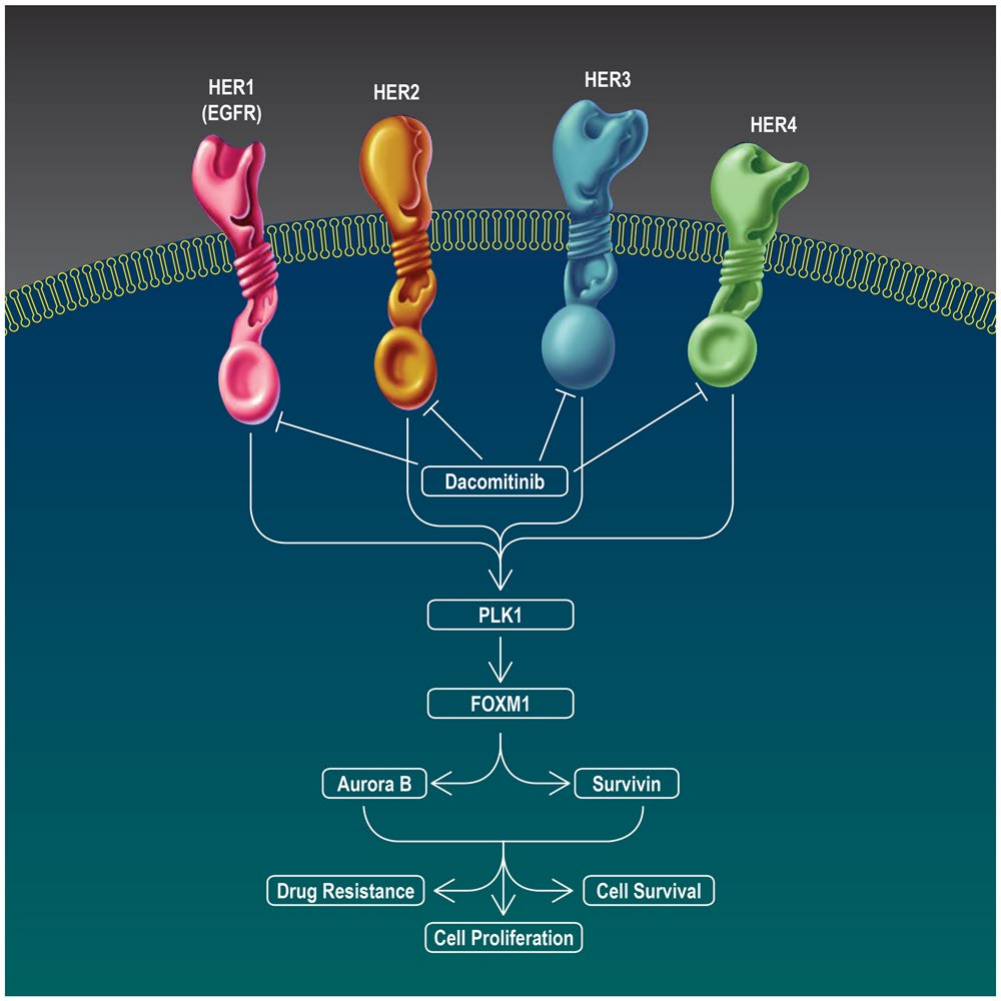
Schematic illustration of the anti-tumour effects of dacomitinib on the EOC cells. Dacomitinib inhibited cell growth, induced apoptosis and restored cisplatin sensitivity through blockade of the PLK1/FOXM1 axis and its downstream targets Aurora B and survivin.

Taken together, the results of the present study provide new insight into the mechanistic activity of dacomitinib through inhibition of the PLK1-FOXM1 axis (Fig. 6). These findings also indicate that dacomitinib-mediated blockade of the ErbB receptors provides advantages over single-targeted ErbB inhibitors and thus offer a novel and promising treatment strategy to address chemoresistance in EOC. Further *in vivo* investigations are warranted to explore the anti-tumour activity of dacomitinib in therapy-resistant EOC.

## Materials and Methods

### Antibodies and chemicals

Antibodies were obtained as follows: Aurora B (Abcam); EGFR, HER2, HER3 (clone 2F12) and HER3-neutralising monoclonal antibody (clone H3.105.5) (Millipore); p-EGFR (Tyr1068), p-HER2 (Tyr1248), p-HER3 (Tyr1289; clone 21D3), p-HER4 (Tyr1284; clone 21A9) and p-PLK1 (Thr210) (Cell Signalling Technology); HER4 (clone C-7), PLK1 (clone F-8), FOXM1 (clone C-20), survivin (clone FL-142) and β-actin (Santa Cruz Biotechnology). Dacomitinib and BI 2536 (a specific inhibitor of polo-like kinase 1) were purchased from Adooq Bioscience (Irvine, CA, USA) and were dissolved in DMSO. The final concentrations of DMSO did not exceed than 0.1% [v/v] in all the treatments. Erlotinib (EGFR small molecule inhibitor) was obtained from Chemietek (Indianapolis, IN, USA). Cetuximab (a ligand-blocking anti-EGFR mAb), trastuzumab (anti-HER2 mAb), cisplatin and doxorubicin (DNA-damaging drugs), paclitaxel (a taxane inhibitor of microtubule disassembly), vincristine (a mitosis-blocking agent), carboplatin (an alkylating agent) and gemcitabine (a nucleoside analogue which inhibits DNA synthesis) were purchased from the pharmacy of Shariati hospital (Tehran, Iran). Poly-hydroxyethylmethacrylate polymer (poly-HEMA) was obtained from Santa Cruz Biotechnology. Recombinant HRGβ-1 was purchased from Peprotech.

### Human ovarian carcinoma cell lines

Human ovarian carcinoma cell lines were obtained from National Cell Bank of Iran (NCBI, Tehran, Iran). These include A2780CP, A2780S, Caov4, OVCAR3 and SKOV3. All the cell lines were authenticated by STR profiling and were routinely checked for mycoplasma infection. Cell cultures were maintained at 37 °C in 5% CO_2_ in a humidified incubator and cultured according to NCBI recommendations.

### Chemosensitivity assay

The EOC cells were seeded at 2 × 10^3^ per well in 96-well plates and treated with the chemotherapeutics for 48 h. Cell viability was determined by MTT assay. Vehicle-treated cells were used as the control group. Chemosensitivity was shown as IC_50_ values calculated from full dose–response curves.

### Median-effect analysis for treatment synergy

To determine the efficacy of combination of cisplatin with the anti-ErbB agents, the cells were treated with 0 to 10 μg/mL of cisplatin and different concentrations of the ErbB inhibitors for 48 h. Survival fractions in each treatment were determined by MTT assay and combination index (CI) was computed using the method developed by Chou and Talalay^78^ and the computer software CalcuSyn (Biosoft, Cambridge, UK). CI < 1, CI = 1 and CI > 1 represent synergism, additive effects and antagonism of the two drugs, respectively.

### Crystal violet staining

The cells were plated at a density of 6 × 10^4^ cells in 6-well plates and treated with the drugs for 48 h. The cultures were then washed with PBS, fixed with ice-cold methanol and stained with crystal violet (0.5% w/v). The images were acquired with an inverted microscope.

### Colony formation assay

Single-cell suspensions were seeded in 6-well plates at a density of 500 cells/well. Once adhered, the cells were treated with the desired concentrations of the drugs for 48 h. The plates were further incubated for 10 d and colonies were stained with 0.5% crystal violet and counted under an inverted microscope. Plating efficiency (PE) and survival fraction (SF) were calculated using the following formula: PE = number of colonies/number of cells seeded; SF = number of colonies/number of cells seeded × PE and plotted graphically to obtain survival curves.

### Anoikis resistance assay

For anoikis evaluation, 96-well plates were coated with poly-HEMA (20 mg/mL in 95% ethanol) and dried in a tissue culture hood. The cells were trypsinised into a single cell suspension and seeded in poly-HEMA-coated plates at a density of 5 × 10^3^ cells/well. The cell suspension cultures were treated with the desired concentrations of the drugs for 48 h. Cell viability was estimated by MTT assay.

### Analysis of gene expression by quantitative reverse transcription-PCR

The quantitative reverse transcription-PCR (qRT-PCR) analysis was performed on a LightCycler 96 instrument (Roche Molecular Diagnostics) using RealQ-PCR Master Mix kit (Ampliqon, Copenhagen, Denmark). The primers used are listed in Supplementary Table 3. The target gene expression levels were normalised to beta-2-microglobulin (*B2M*) levels in the same reaction. For calculations, 2^−ΔΔC^_T_ formula was used, with ΔΔC_T_ = (C_T_ *target* − C_T_ *B2M*) experimental sample – (C_T_ *target* − C_T_ *B2M*) control samples, where C_T_ is cycle threshold^79^.

### Western blot analysis

The cells were lysed with RIPA lysis buffer (50 mM Tris-HCl, pH 8.0, 150 mM NaCl, 1.0% NP-40, 0.5% sodium deoxycholate and 0.1% SDS) containing protease and phosphatase inhibitors (Roche Molecular Biochemicals). Protein concentration was determined using the Bradford assay. A total of 50 to 100 μg of protein were separated by SDS-PAGE and transferred onto PVDF membranes (Membrane Solutions, TX, USA). Membranes were blocked and blotted with the relevant antibodies. Horseradish peroxidase-conjugated secondary antibodies were detected with a BM chemiluminescence detection kit (Roche Molecular Biochemicals). β-actin was used as the loading control. All antibody dilutions were 1:500 except for the β-actin antibody, which was used at a dilution of 1:5000.

### Annexin V/PI staining

Cells were stained with PI and FITC-conjugated Annexin V, as provided by Annexin-V-FLUOS Staining Kit (Roche Diagnostics) according to the manufacturer’s instructions. The results were analysed using a Partec PAS III flow cytometer (Partec GmbH) and WindowsTM FloMax software (Partec).

### Cell migration and invasion

Transwell cell migration and invasion assays were carried out as described earlier^80^.

### Statistical analysis

All data were evaluated in triplicate against vehicle-treated control cells and collected from three independent experiments. Data were graphed and analysed by GraphPad Prism Software 7.0a using one-way ANOVA and the unpaired two-tailed Student’s *t*-test. All data are presented as mean ± standard deviation (SD).

## Electronic supplementary material

Supplementary Info
