# Supplementary material for: Dacomitinib, a pan-inhibitor of ErbB receptors, suppresses growth and invasive capacity of chemoresistant ovarian carcinoma cells
Source: Sci Rep. 2017 Jun 23;7:4204. doi: 10.1038/s41598-017-04147-0 (PMC5482808; doi:10.1038/s41598-017-04147-0)
Supplement: Supplementary file 1 — Supplementary Info [file 41598_2017_4147_MOESM1_ESM.pdf]

Dacomitinib, a pan-inhibitor of ErbB receptors, suppresses growth and invasive capacity of chemoresistant ovarian carcinoma cells

Majid Momeny[1,+], Ghazaleh Zarrinrad[1,+], Farima Moghaddaskho[1], Arash Poursheikhani[2], Ghazaleh Sankanian[1], Azam Zaghal[1], Shahab Mirshahvaladi[3], Fatemeh Esmaeili[2], Haniyeh Eyvani[1], Farinaz Barghi[1], Zahra Sabourinejad[1], Zivar Alishahi[2], Hassan Yousefi[2], Reza Ghasemi[4], Leila Dardaei[5], Davood Bashash[6], Bahram Chahardouli[1], Ahmad R. Dehpour[7], Javad Tavakkoly-Bazzaz[2], Kamran Alimoghaddam[1], Ardeshir Ghavamzadeh[1], Seyed H. Ghaffari[1,*]

[1]Haematology/Oncology and Stem Cell Transplantation Research Centre, Shariati Hospital, School of Medicine, Tehran University of Medical Sciences, Tehran, Iran

[2]Department of Medical Genetics, School of Medicine, Tehran University of Medical Sciences, Tehran, Iran

[3]Department of Molecular Systems Biology, Cell Science Research Centre, Royan Institute for Stem Cell Biology and Technology, Tehran, Iran

[4]Section of Stem Cell Biology, Division of Oncology, Department of Medicine, Washington University in Saint Louis, MO, USA

[5]Massachusetts General Hospital Cancer Centre, Charlestown, MA, USA

[6]Department of Haematology and Blood Banking, Faculty of Allied Medicine, Shahid Beheshti University of Medical Sciences, Tehran, Iran

[7]Experimental Medicine Research Centre, Tehran University of Medical Sciences, Tehran, Iran

[+]These authors contribute equally to this work

*Correspondence to: Seyed H. Ghaffari, email: shghaffari200@yahoo.com

Keywords: Epithelial ovarian cancer; Chemoresistance; ErbB family; Dacomitinib

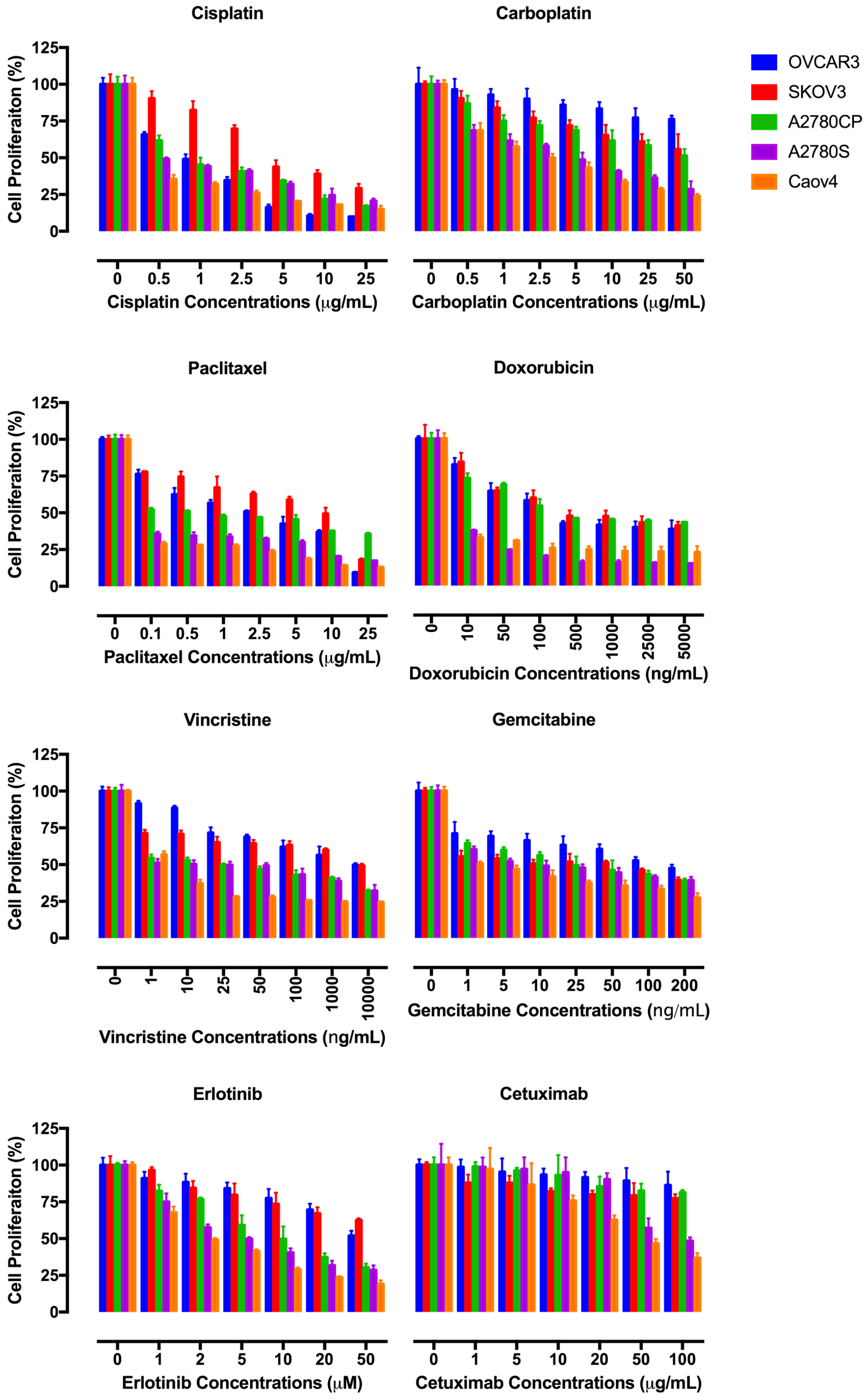

Supplementary Fig. 1

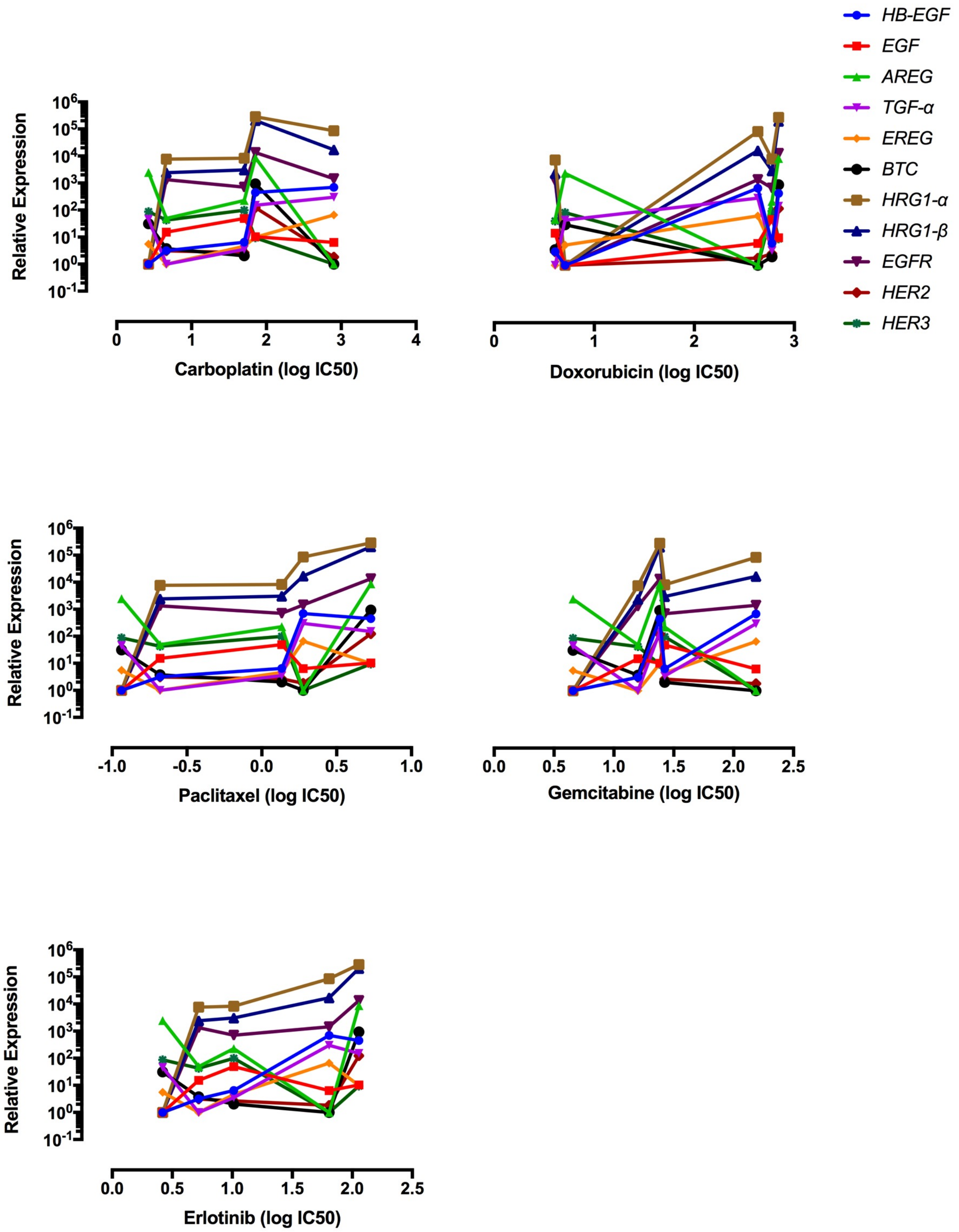

Supplementary Fig. 2

Supplementary Table 1. Combination index (CI) and dose reduction index (DRI) of erlotinib-cisplatin combination in OVCAR3, SKOV3 and A2780CP cells. DRI represents the order of magnitude of dose reduction that is allowed in combination for a given degree of effect as compared with the dose of each drug alone. ''fa'' denotes fraction affected.

**Concentrations**		**fa**	**CI**	**DRI**	
**Erlotinib (µM)**	Cisplatin (µg/mL)			Erlotinib	Cisplatin
		**OVCAR3**			
**5**	0.1	0.41	3.311	0.327	3.994
**5**	0.5	0.55	1.856	0.807	1.622
**5**	1	0.61	1.707	1.224	1.124
**5**	2.5	0.74	1.297	3.388	0.998
**5**	5	0.86	0.821	12.106	1.354
**5**	10	0.89	1.137	18.018	0.925
		**SKOV3**			
**5**	0.1	0.28	1.785	0.695	2.885
**5**	0.5	0.33	2.206	0.974	0.848
**5**	1	0.41	2.004	1.569	0.732
**5**	2.5	0.63	0.974	5.610	1.257
**5**	5	0.74	0.763	11.758	1.465
**5**	10	0.76	1.193	13.960	0.891
		**A2780CP**			
**5**	0.1	0.44	3.001	0.336	45.756
**5**	0.5	0.49	2.440	0.425	11.089
**5**	1	0.51	2.385	0.452	5.821
**5**	2.5	0.56	2.110	0.570	2.810
**5**	5	0.6	2.051	0.693	1.645
**5**	10	0.66	1.998	0.948	1.060

Supplementary Table 2. Combination index (CI) and dose reduction index (DRI) of dacomitinib-cisplatin combination in OVCAR3, SKOV3 and A2780CP cells.

Concentrations		fa	CI	DRI	
**Dacomitinib (µM)**	Cisplatin (µg/mL)			Dacomitinib	Cisplatin
		**OVCAR3**			
**5**	0.1	0.75	0.315	3.53	31.24
**5**	0.5	0.76	0.407	3.90	6.67
**5**	1	0.81	0.405	5.73	4.33
**5**	2.5	0.87	0.436	11.91	2.84
**5**	5	0.92	0.464	25.24	2.36
**5**	10	0.92	0.873	25.87	1.20
		**SKOV3**			
**5**	0.1	0.46	0.294	3.904	26.283
**5**	0.5	0.49	0.394	4.623	5.628
**5**	1	0.59	0.383	8.983	3.681
**5**	2.5	0.78	0.397	39.731	2.686
**5**	5	0.86	0.526	98.399	1.938
**5**	10	0.87	0.983	113.560	1.027
		**A2780CP**			
**5**	0.1	0.75	0.460	2.320	34.853
**5**	0.5	0.76	0.536	2.498	7.375
**5**	1	0.81	0.528	3.262	4.516
**5**	2.5	0.87	0.548	5.570	2.711
**5**	5	0.92	0.589	9.671	2.061
**5**	10	0.92	1.058	9.851	1.045

Supplementary Table 3. Nucleotide sequences of the primers used for qRT-PCR

Gene	Accession	Forward Primer	Reverse Primer	Amplicon
B2M	NM_004048	GATGAGTATGCCTGCCGTGT	CTGCTTACATGTCTCGATCCCA	79
CCNB1	NM_031966	AATAAGGCGAAGATCAACATGGC	TTTGTTACCAATGTCCCCAAGAG	111
CDK1	NM_001786	AAACTACAGGTCAAGTGGTAGCC	TCCTGCATAAGCACATCCTGA	148
BIRC5	NM_001168	CCAGATGACGACCCCATAGAG	TTGTTGGTTTCCTTTGCAATTTT	152
CDC25C	NM_001790	TCTACGGAACTCTTCTCATCCAC	TCCAGGAGCAGGTTTAACATTTT	98
FOXM1	NM_202002	ATAGCAAGCGAGTCCGCATT	AGCAGCACTGATAAACAAAGAAAGA	151
AURKB	NM_004217	GCTCTCCTCCCCCTTTCTCT	TGTGAAGTGCCGCGTTAAGA	245
HBEGF	NM_001945	ATCGTGGGGCTTCTCATGTTT	TTAGTCATGCCCAACTTCACTTT	86
AREG	NM_001657	GAGCCGACTATGACTACTCAGA	TCACTTTCCGTCTTGTTTTGGG	121
TGFA	NM_003236	AGGTCCGAAAACACTGTGAGT	AGCAAGCGGTTCTTCCCTTC	87
EREG	NM_001432	GTGATTCCATCATGTATCCCAGG	GCCATTCATGTCAGAGCTACACT	120
BTC	NM_001729	CCTGGGTCTAGTGATCCTTCA	CTTTCCGCTTTGATTGTGTGG	131
ZEB1	NM_001128128	TGTGGTAGAAACAAATTCAGATTCA	GCCCTTCCTTTCCTGTGTCA	200
ZEB2	NM_014795	TGGTCCAGAAGAAATGAAGGAAGA	GTCACTGCGCTGAAGGTACT	190
CDH2	NM_001792	AGGCTTCTGGTGAAATCGCA	TGCAGTTGCTAAACTTCACATTG	120
BCL2	NM_000633	CAGGATAACGGAGGCTGGGATG	TTCACTTGTGGCCCAGATAGG	154
EGFR	NM_005228	TATTGATCGGGAGAGCCGGA	TCGTGCCTTGGCAAACTTTC	145
HER2	NM_004448	TGTGACTGCCTGTCCCTACAA	CCAGACCATAGCACACTCGG	152
HER3	NM_001982	GGTGATGGGGAACCTTGAGAT	CTGTCACTTCTCGAATCCACTG	80
EGF	NM_001963	TGTCCACGCAATGTGTCTGAA	CATTATCGGGTGAGGAACAACC	133
HRG1-α	NM_013964	AAACCAAGAAAAGGCGGAGGAGCT	GAGGGCGATGCAGATGCCGG	70
HRG1-β	NM_013956	GCCAGCTTCTACAAGCATCTTGGGA	GGAGGGCGATGCAGATGCCG	97
HER4	NM_005235	GTTCAGGATGTGGACGTTGC	GCCTCCAGCACATTCTCGAT	164

Supplementary legends

Supplementary Fig. 1: Chemosensitivity of the EOC cell lines to various drugs was determined by MTT assay and expressed as percentage of the control cells. The data represent mean ± SD of three independent experiments, each performed in triplicate.

Supplementary Fig. 2: No correlation between the expression of the ErbB family and resistance to carboplatin, paclitaxel, doxorubicin, gemcitabine and erlotinib were found in the EOC cell line models.
